# Signal propagation and neuronal avalanches analysis in networks of formal neurons

**DOI:** 10.1186/1471-2202-12-S1-P172

**Published:** 2011-07-18

**Authors:** Mauricio Girardi-Schappo, Marcelo HR Tragtenberg, Osame Kinouchi

**Affiliations:** 1 Departamento de Física, Universidade Federal de Santa Catarina, Florianópolis, SC, 88040-970, Brazil; 2 Faculdade de Filosofia, Ciências e Letras de Ribeirão Preto, Universidade de São Paulo, Ribeirão Preto, SP, Brazil

## 

To study neurons with computational tools, one may call upon, at least, two different approaches: (i) Hodgkin-Huxley like neurons [1] (i.e. biological neurons) and (ii) formal neurons (e.g. Hindmarsh-Rose (HR) model [2], Kinouchi-Tragtenberg (KT) model [3], etc). Formal neurons may be represented by ordinary differential equations (e.g. HR), or by maps, which are dynamical systems with continuous state variables and discrete time dynamics (e.g. KT). A few maps had been proposed to describe neurons [3-10]. Such maps provide one with a number of computational advantages [10], since there is no need to set any precision on the integration variable, which leads to better performance in the calculations.

An extended KT neuron model, here called KTz model, has been studied in [4] and [5], may be supplied with a Chemical Synapse Map (CSM) in order to study interacting neurons in a lattice, in the framework of coupled map lattices. KTz model presents most of the standard behavior of excitable cells like fast spiking, regular spiking, bursting, plateau action potentials and adaptation phenomena, and the CSM is in good agreement with some standard functions used to model post-synaptic currents, like the alpha function or the double-exponential function [4]. Preliminary results indicate *antiferromagnetic* oscillatory behavior or *plane wave* behavior in KTz neurons coupled with inhibitory CSM on a square lattice.

Besides, many systems in nature are characterized by complex behavior where large cascades of events, named avalanches, unpredictably alternate with periods of little activity (e.g. snow avalanches, earthquakes, etc). Avalanches are described by power law distributions and when the branching parameter equals to unity, the system is said to be a self-organized critical (SOC) system [13]. These have been observed for neuronal activity in vitro [11,12]. And since both SOC systems and neuronal activity show large variability, long-term stability and memory capabilities, networks of neurons have been proposed to be SOC systems. This hypothesis was tested in [13], where they made comparisons among in vivo recordings using Local Field Potentials in three macaque monkeys performing a short term memory task and three different well-established subsampled SOC models (e.g. Sandpile model, Random Neighbour Sandpile model and Forest Fire model). Some similar comparison has been done in [14] with in vivo data from fourteen rats and a cellular automaton developed by the authors.

We claim that still no simulation has been made to detect whether formal or realistic neuron models can evolve naturally to a SOC state, in a full or subsampled network. Our simulations are made with KTz model, which is a formal neuron, but keeps the usual behaviors of living cells, connected through CSM on a square lattice. We divided the work into two parts: (i) the analysis of network itself and how it evolves with time from a given initial state, varying its parameters and (ii) the analysis of the data generated by a network of silent cells, stimulated at random sites, trying to resemble the SOC models above. We compare these second part results with the experimental ones presented in [11-13].
